# *Salmonella enterica* Serovar Typhimurium and *Escherichia coli* Survival in Estuarine Bank Sediments

**DOI:** 10.3390/ijerph15112597

**Published:** 2018-11-21

**Authors:** Mahbubul H. Siddiqee, Rebekah Henry, Rebecca Coulthard, Christelle Schang, Richard Williamson, Rhys Coleman, Graham Rooney, Ana Deletic, David McCarthy

**Affiliations:** 1 Environmental and Public Health Microbiology Laboratory (EPHM LAB), Department of Civil Engineering, Monash University, Melbourne, VIC-3168, Australia; mhsiddiqee@gmail.com (M.H.S.); Rebekah.Henry@monash.edu (R.H.); Rebecca.Coulthard@wsp.com (R.C.); Christelle.Schang@monash.edu (C.S.); Richard.Williamson@monash.edu (R.W.); a.deletic@unsw.edu.au (A.D.); 2 Molecular and Environmental Microbiology Laboratory (MEM LAB), Department of Mathematics and Natural Sciences, BRAC University, Dhaka 1212, Bangladesh; 3 Melbourne Water Corporation, Docklands, VIC-3008, Australia; Rhys.Coleman@melbournewater.com.au (R.C.); grahamrrooney@gmail.com (G.R.)

**Keywords:** fecal indicator, fecal pathogen, waterborne pathogens, recreational risks, QMRA

## Abstract

Estuarine bank sediments have the potential to support the survival and growth of fecal indicator organisms, including *Escherichia coli*. However, survival of fecal pathogens in estuarine sediments is not well researched and therefore remains a significant knowledge gap regarding public health risks in estuaries. In this study, simultaneous survival of *Escherichia coli* and a fecal pathogen, *Salmonella enterica* serovar Typhimurium, was studied for 21 days in estuarine bank sediment microcosms. Observed growth patterns for both organisms were comparable under four simulated scenarios; for continuous-desiccation, extended-desiccation, periodic-inundation, and continuous-inundation systems, logarithmic decay coefficients were 1.54/day, 1.51/day, 0.14/day, and 0.20/day, respectively, for *E. coli*, and 1.72/day, 1.64/day, 0.21/day, and 0.24/day for *S.* Typhimurium. Re-wetting of continuous-desiccated systems resulted in potential re-growth, suggesting survival under moisture-limited conditions. Key findings from this study include: (i) Bank sediments can potentially support human pathogens (*S.* Typhimurium), (ii) inundation levels influence the survival of fecal bacteria in estuarine bank sediments, and (iii) comparable survival rates of *S.* Typhimurium and *E. coli* implies the latter could be a reliable fecal indicator in urban estuaries. The results from this study will help select suitable monitoring and management strategies for safer recreational activities in urban estuaries.

## 1. Introduction

Urban estuaries deliver multiple benefits to communities, including access to aquatic recreation, amenity, transport, and supporting cultural traditions. As a result, 22 of the largest cities in the world are located adjacent to estuaries [1]. However, community benefits from estuaries are under increasing threat from pollution due to progressive urbanization and population growth [2]. Fecal contamination is one of the leading threats to public health in these systems, via exposure to harmful organisms during aquatic recreation [3,4,5]. 

Monitoring of public health risks in estuaries has historically relied on fecal indicator organisms, such as *Escherichia coli* and *Enterococcus* spp.—principally due to the practical limitations and costs associated with measuring pathogens. In many cases, significant correlations have been observed between health outcomes (i.e., illness rates) and indicator organism concentrations in marine waters and some freshwater systems [6,7]. However, there is a paucity of epidemiological studies for estuarine environments [8], meaning that the link between indicator organisms and human health outcomes in these systems is more uncertain.

There are an increasing number of studies that show that fecal indicators can survive and grow in estuarine systems, especially in their sediments where they are protected from competition and predation and have a rich nutrient supply [9]. For example, Solo-Gabriele et al. (2000) and Desmarais et al. (2002) found that coastal bank sediments are a suitable habitat for the survival and growth of *E. coli*. Further, Solo-Gabriele et al. (2000) found that that growth of *E. coli* was a function of soil moisture content and hence, different soil moisture scenarios (as a result of tidal fluctuation) could potentially exert various degrees of stress, leading to differential survival of fecal organisms. At the same time, the survival potential of pathogens in these sediments may indeed be different to that of the indicator organism, *E. coli*. Moreover, the survival of fecal organisms in the bank sediments of estuaries in temperate climates is not well understood. 

There are only a handful of studies that have explored the survival of pathogens in estuarine systems, and even fewer which have focused on estuarine sediments. Indeed, there are only two reported studies which have explored pathogen survival within the sediments of estuaries [9,10]. This lack of pathogen-survival studies in these types of systems is especially important considering the salient features of estuarine bank sediment as opposed to other soils in terms of its particle size distribution, organic content, composition of minerals, and organic matters [11]. With others having demonstrated extended survival and resuspension [12,13] of fecal indicator organisms from the bank sediments, this paucity in pathogen-survival data leaves a critical knowledge gap regarding the reliability of these indicators for predicting public health risk in these systems.

This study aims to determine: (a) Whether different sections of tidally-influenced estuarine bank sediments (fully inundated, periodically inundated, occasionally inundated, and fully desiccated) can support the extended survival of a human pathogen (*Salmonella enterica* serovar Typhimurium), and (b) whether the survival of a commonly used fecal contamination indicator (*E. coli*) is comparable to *Salmonella* Typhimurium. *Salmonella* Typhimurium is recommended by the US EPA as a suitable pathogen for water-based monitoring [14] and it is also responsible for the majority of *Salmonella*-related infections (nearly 80% in Australia) [15].

## 2. Materials and Methods

### 2.1. Bacterial Strains and Inoculum Development

In this study, *Escherichia coli* K1 was used as a model indicator organism and *S.* Typhimurium NVSL 6993 as the model enteric pathogen. *E. coli* and *S.* Typhimurium were subcultured from glycerol stock (maintained at −80 °C) onto Eosine-Methylene-Blue (EMB) (Oxoid, UK) agar and Xylose-Lysine-Deoxcolate (XLD) (Oxoid, UK) agar, respectively. Plate cultures were incubated overnight at 37 °C. Single colonies were isolated and transferred to 150 mL LB broth and incubated for 12 h at 37 °C with shaking (60 rpm). The cultures were pelleted at 4000 × *g* for 15 min (at 10 °C). Supernatants were discarded, and the pellets resuspended in 5 mL Phosphate Buffered Saline (PBS) (1×; pH 7.4). Pelleting was repeated, the supernatant discarded, and the final pellet resuspended in 5 mL PBS (1×; pH 7.4).

### 2.2. Collection and Processing of Bank Sediment

**Figure A1:**
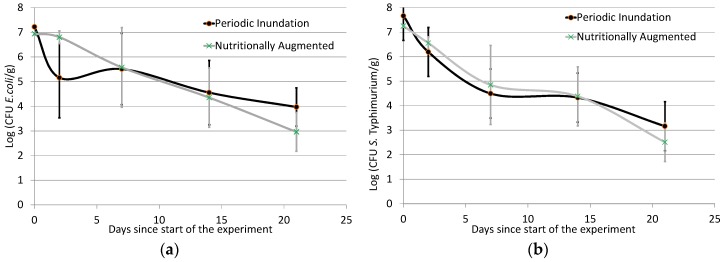
Influence of augmented nutrition on survival of (**a**) *E. coli* K1 and (**b**) *S.* Typhimurium NVSL 6993 (error bars represent standard deviations around the mean values for each of the configurations).

The top 8 cm layer of bank sediments (20 kg) were collected at low tide from the Yarra River estuary at South Yarra (Victoria, Australia). The sediment was mixed to ensure homogeneity followed by sterilization by autoclaving before cooling it to room temperature. Since the main focus of this study was to test the influence of inundation scenarios, autoclaving of the bank sediment before inoculation with the two test microorganisms was done. This sterilization was essential to ensure that the survival rates of *E. coli* and *S.* Typhimurium were not influenced by predation, competition, or parasitism by native microorganisms present in natural sediments, which are known to have differential effects on these two organisms in different scenarios [16,17,18]. The influence of autoclaving on the nutritional properties of the sediment was tested prior to experimentation; no significant change in concentrations of total phosphorus (TP) and total dissolved nitrogen (TN) was observed when measured according to the Australian Laboratory Handbook of Soil and Water Chemical Methods, 1992 (see Figure A1 and Table A1). Changes in particle size distribution were also investigated using the electro-resistance counting method [19] and no detectable change was observed between autoclaved and non-autoclaved sediments. 

The sterilized sediment was inoculated with both bacterial strains to reach a final concentration of 10^7^ g^−1^ (wet sediment) cells. After mixing, the inoculated sediment was distributed into sterilized plastic containers by sub-sampling 10 × 100 g aliquots into 14 open plastic containers, using a randomization approach [20], to achieve a 3 cm thick layer consisting of 1 kg of sediment.

### 2.3. Experimental Matrix

Four experimental configurations were investigated in triplicate to represent four typical exposed tidally influenced bank sediment areas (Table 1): (i) continuous desiccation (CD), which received no inundation and represented bank sediment above the high tide mark, which does not receive any moisture from inundation; (ii) extended desiccation (ED), no inundation for the first seven days followed by periodic inundation after this period and represented the section of bank sediment that is only inundated during extreme conditions (including high flow periods and/environmental flow releases); (iii) periodically inundated (PI), representing tidally influenced sediment between high and low tide marks, which received the normal tidal of the condition of the Yarra estuary with a 12.4 h inundation cycle; and (iv) continuously inundated (CI) systems, which were permanently inundated with water and represent bank sediments just below the low tide mark. A non-inoculated control (NI) was used to monitor for environmental contamination that may occur during the experiment as a result of the open system design. Sediment containers were faced toward the sun with a slope of 7° (same as observed in the bank at the South Yarra site from where the sediment was collected).

### 2.4. Experimental Procedure

**Figure 1:**
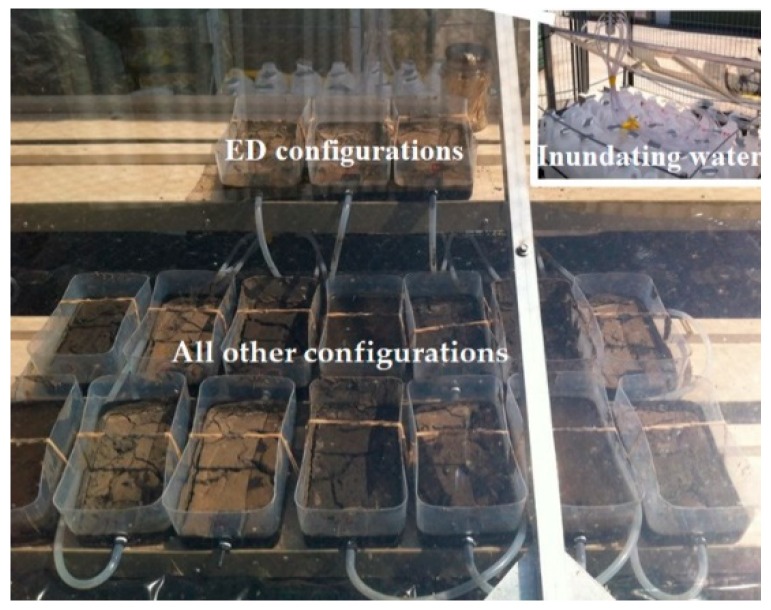
Outdoor experimental set up for the bank sediment survival study, which is covered by a transparent Perspex sheet; ED configurations placed on a higher platform and all the others on the lower platforms. Bottles containing water for inundating sediments (see upper right corner) were located on a vertically moving platform.

All configurations were placed outdoor (Figure 1) under a Perspex sheet to protect the configurations from rain and direct fecal deposition by local wildlife. ED configurations were placed on a high platform to ensure shorter exposure to inundations compared to the PI configurations. The remaining configurations were placed on the low platforms in a randomized manner to account for differences related to spatial distribution. ED, PI, and NI containers were connected with silicon hoses to sterile plastic bottles (autoclaved) containing 1 L (each) of sterile semi-synthetic river water; representing water quality parameters of the Yarra River during a similar dry weather period at the same location. Data from a previous project (data not shown) was used to calculate target TP, TN, and total suspended solid (TSS) concentrations (0.09 mgL^−1^, 0.91 mgL^−1^, and 23 mgL^−1^, respectively). The stock semi-synthetic water was made using autoclaved bed sediment (collected from the river at South Yarra) to produce a final TSS concentration of 23 mgL^−1^. As sediment alone was unable to reproduce the target TN and TP concentrations, inorganic salts (KH_2_PO_4_ for TP and a mixture of KNO_3_, NH_4_Cl, and C_6_H_5_O_2_N for TN) were added as outlined by Bratieres et al. (2008) to achieve the target concentrations [21]. The stock water was stored in room temperature and periodically tested to ensure sterility throughout experimentation. The water containers (all containing 1 L each) were kept on a platform, which had a periodic vertical movement mimicking the tidal fluctuations of the Yarra River (12.4 h cycle) covering the intended sediment configurations. The inundating water was replaced every week. 

The survival experiment was conducted over 21 days. The first three days of the experiment coincided with a heat wave experienced in Melbourne; on each day, the maximum atmospheric temperature exceeded 40 °C. The average daily maximum temperature during this experiment was 29.4 °C and the average solar radiation was 25.6 MJ/m^2^ (BOM, Australia, 2014). 

### 2.5. Collection and Processing of Samples

A total of 6 g of top layer sediment was sampled (in four sub-samples) from each configuration on Days 0, 2, 7, 14, and 21. Further samples were collected from PI and ED on Day 1. The four sub-samples were homogeneously mixed to make a uniform mixture. Moisture content measurements were conducted on ~1.5 g of sediment following Standards Australia, (2005) [22]. For culture-based assays, ~1.5 g of sediment was dissolved in 25 mL sterile de-ionized water and mixed for 1 min at 120 shakes min^−1^ to dislodge particle-bound bacteria. The homogenized mix was pelleted at 400× *g* for 5 min. From the supernatant, a single 5 mL aliquot was diluted in 5 mL PBS (1×; pH 7.4) and vortexed for 3 s. The diluted supernatants were then further diluted to a final concentration of 10^−4^ in PBS (1×; pH 7.4). 

### 2.6. Enumeration of E. coli and S. Typhimurium and Calculation of Die-Off Rates

A volume of 100 μL was plated, in triplicate, from the 10^−3^ and 10^−4^ dilutions on EMB agar for *E. coli*, and on XLD agar for *S.* Typhimurium for culturable counts as two differential media are needed to monitor two different organisms in the same system [23,24]. Plate counts were converted to cfu/g (dry weight) based on the relevant dilution factor and corresponding moisture content. Colony counts were then log-transformed and used to construct a line of best fit and the gradient of this line was used to estimate the die-off rate [25]. In cases where no colony was observed in any of the triplicate plates, data points were replaced with half of the detectable limit (i.e., 0.5 cfu/plate [26]). Data points that fluctuated around the lowest detectable limit were not used to estimate die-off rates. Colony counts of below 30 cfu/plate were considered for calculation of die-off rates, with possible uncertainties taken into consideration during data analysis and interpretation. The overall die-off rates were calculated as the logarithmic difference of initial and final numbers divided by the number of days elapsed.

## 3. Results and Discussion

### 3.1. The Impact of Experimental Design

Preliminary investigations demonstrated the insignificant impact that autoclaving had on particle size distributions and nutrient contents. Furthermore, other analyses (Appendix A and Appendix B) demonstrated that the process of autoclaving did not significantly impact microbial survival through the re-release of nutrients from dead microorganisms. However, since the experiment was conducted in an outdoor environment, it was influenced by the prevailing atmospheric conditions. 

**Figure 2:**
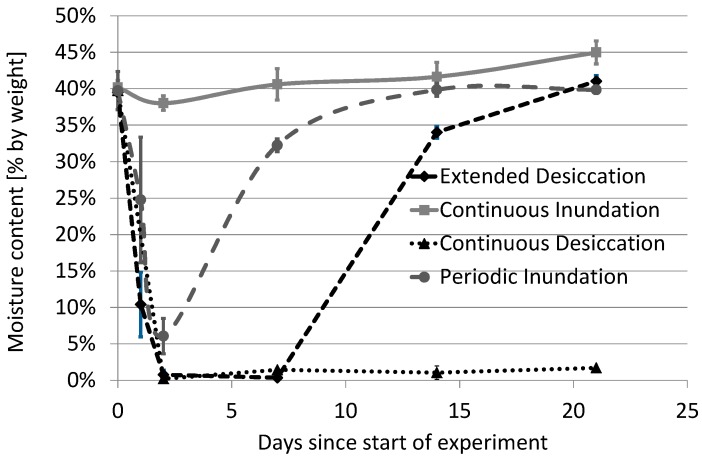
Levels of moisture content in four test sediment configurations during the experiment (error bars represent standard deviations around the mean values for each of the configurations).

The initial phase of the experiment coincided with high atmospheric temperatures (>40 °C) and solar radiation, both known to reduce survival of *E. coli* and *S.* Typhimurium [11,26,27]. During this initial period, a sharp decrease in moisture level was observed in configurations without inundation (CD and ED). In fact, on Day 2, there was almost no moisture remaining in these systems (Figure 2). Overall, the moisture content readings of different configurations were reduced significantly (from 41% to as low as 10%) in all configurations, except for the CI system. 

Another by-product of the heat wave was an increase in TP and TN concentration in the sediment and inundating water (Table A1 and Table A2). However, since the water was replaced weekly, exposure to the increased nutrient levels was not persistent. Furthermore, the concentration of TN and TP within the sediment (0.21 and 0.81 mg/g, respectively, at the beginning) showed very little change (up to 0.4 mg/g for TP and up to 1.4 mg/g for TN) across the experimental period. Thus, it can be assumed that the alteration in water column concentrations did not have a significant effect on sediment survival rates. 

### 3.2. Survival of E. coli and S. Typhimurium

**Figure 3:**
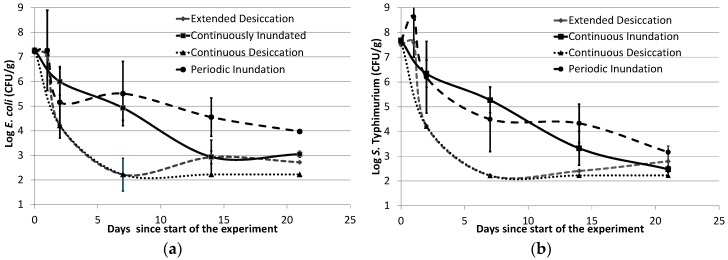
Survival of the test organisms; (**a**) *E. coli* K1 densities (cfu/g dw) and (**b**) *S.* Typhimurium NVSL 6993 densities (cfu/g dw) in the four experimental configurations (error bars represent standard deviations around the mean values of the replicates tested).

The results of the survival study indicate that both *E. coli* and *S.* Typhimurium are able to survive for up to 21 days in all three configurations that received moisture (ED, PI, and CI). Both microorganisms withstood the first 24 h of the experiment after sediment inoculation, which was evident from the culture counts of the two tested configurations (PI and ED; Figure 3). Notably, even though the atmospheric temperature reached 43 °C within a few hours of inoculation, *E. coli* counts increased marginally, and *S.* Typhimurium counts increased nearly 10 times of the initial number. This initial growth could reflect the fact that fecal microorganisms are adapted to animal gut environments (37 °C for mammals and 42 °C for birds). Sediment properties that are widely known to influence bacterial survival include available nutrients [28], organic matter [29], particle size [30], and clay content [31]. Among these, estuarine bank sediment is generally known to have finer particles [32,33], as well as higher organic matter content [34], which can offer several advantages, such as protection from UV light [35,36] and nutritional support. Therefore, it is perhaps not surprising to see extended survival of fecal microorganisms, including the potential pathogen, in estuarine banks even in unfavorable weather conditions. This, in turn, suggests that in the absence of other stressors, *S.* Typhimurium can probably grow in estuarine bank sediments in high-temperature conditions when moisture is available and potentially serve as a source of this pathogen to the water column if re-suspended. 

The initial phase of growth in the first 24 h was followed by a sharp die-off, consistent across organisms and configurations (Figure 3). The CD system had the highest die-off rates for both types of bacteria during the experiment; 1.54/day for *E. coli* and 1.72/day for *S.* Typhimurium. Since significant losses in soil moisture took place during the early phase of the experiment (moisture content decreased from 40% to <1%), these very fast die-off rates were consistent with the literature [11,37]. In addition, high die-off rates might have been facilitated by very high air temperatures [38] coupled with high radiation levels [39]. 

The two test organisms in the ED systems behaved similarly to the CD systems in the first week of the experiment (Figure 3). The die-off rate for *E. coli* was calculated to be 1.51/day and for *S.* Typhimurium it was 1.64/day. Interestingly, when inundation was restored to these systems after Day 7 (although the ED system received a shorter duration of inundation compared to PI counterparts), an increase in moisture content coincided with the detection of *E. coli* colonies on both Days 14 and 21 (in two of the three replicates on both days). *S.* Typhimurium was also detected on Day 21 in one of the replicate boxes while the NI controls remained negative. An increase in moisture level due to inundation restoration might have influenced this regrowth/recurrence of both test organisms.

The detection of culturable bacteria after a period of drying indicates that both bacteria may survive either in dormant form or at least survive in very low densities. It is important to note that although above the limit of detection, the colony counts were below 30 cfu/plate, and therefore may have higher associated uncertainties. However, colonies were reproducibly isolated within the replicate, suggesting a level of survival was possible. Dormancy and subsequent regrowth of *S.* Typhimurium upon increasing moisture has previously been reported [40]. Also, desiccation has been shown for both *E. coli* and *S.* Typhimurium to significantly enhance resistance towards environmental stressors, including high temperature [41,42]. This could perhaps partly explain the recurrence of these two organisms in the ED systems. Therefore, it is possible that bank sediments, which only receive moisture very intermittently (i.e., bank sediments above the usual high-tide mark, which are inundated only during king tides or high flows) could support the growth of viable fecal pathogens that, if washed, could get resuspended back into the water column [43]. Resuspension could also occur due to other natural and anthropogenic activities, like storms, floods, recreational activities, and commercial dredging, which have all been known to cause resuspension of sediment borne bacteria and causing elevated levels of fecal organisms [44,45]. Therefore, extended survival of potential fecal pathogens, like *S.* Typhimurium, in estuarine bank sediment could comprise a potential human health risk.

PI systems had higher levels of survival for both *E. coli* and *S.* Typhimurium compared to the desiccated CD and ED systems (Figure 3). For PI, the decay rate of *E. coli* was 0.14/day, while a slightly higher decay rate was observed for *S.* Typhimurium (0.21/day). The decrease in cell counts after Day 1 may be associated with very high air temperatures and solar radiation, which also resulted in a very low moisture content (close to 5%; Figure 2). It has previously been reported that Gram-negative bacteria require a moisture content of 93% or more for optimal growth [46]. However, despite some variation among the three biological replicates for the PI configurations, it was evident that the scenario still supported the survival of both test organisms for up to three weeks. Therefore, under more favorable conditions (lower atmospheric temperature, lower solar radiation, and higher moisture), it is likely that survival of these fecal organisms (including potential pathogens) would be higher. 

Survival rates of *E. coli* and *S.* Typhimurium in the PI configuration were found to be comparable. This contradicts the notion that fecal pathogens cannot survive in estuarine sediments, and suggests that *E. coli* could be a reliable indicator of this pathogen in this scenario. Furthermore, the extended survival of a potential fecal pathogen in the tidally influenced zone of an estuarine bank could have human health implications. This is especially the case for bank sediments, where natural and anthropogenic activities (including tide, storm, flood, recreation, dredging, etc.) can readily cause resuspension into the water column [10,45,47].

The CI systems showed slightly higher die-off rates when compared to PI (0.20/day for *E. coli* and 0.24/day for *S.* Typhimurium). It was interesting to see higher die-off rates compared to those of PI, especially since the organisms in the CI systems were exposed to higher moisture levels and reduced solar irradiation. However, the water used for inundating the experimental containers was stagnant in the CI configurations, which led to some interference due to the formation of algae after the first week, and this may have resulted in a faster die-off. The algal growth resulted in lower TP and TN concentrations in the inundating water in the CI systems compared to the other configurations (Table A2). 

In the estuarine context, better survival of *E. coli* in areas of coastal bank sediment with intermittent drying effect compared to continuously moist sediment has previously been reported by Solo-Gabriele et al. (2000). They argued that this better survival was due to having a better competitive advantage over the predators existing in natural sediment. Although our study attempted to exclude the biotic factors by sterilizing the soil beforehand, the CI systems with algal interference supports the idea that algal bloom (which could occur in estuarine systems) could potentially reduce the survival of fecal organisms in estuarine bank sediments. In this scenario, even under these algae-influenced conditions, the survival rates of both organisms were again comparable, reinforcing the ability of *E. coli* to represent *S.* Typhimurium in complex systems, including those with algae blooms. It is also important to note that apart from the CI systems, no other configurations showed any sign of algal interference.

This study was conducted under open atmospheric conditions, which led to some challenges, including changing TP and TN concentrations. However, other studies have demonstrated that increases in TP does not necessarily impact the survival of fecal organisms, like *E. coli* [48,49]. Likewise, increased levels of organic or inorganic nitrogen do not necessarily impact the survival of fecal bacteria in ambient waters [48]. Therefore, it can be assumed that the changes in TP and TN concentrations did not significantly influence the die-off rates of the test organisms. In fact, conducting the study in an ambient environment allowed a unique opportunity to explore the comparative survival of these organisms under highly unfavorable conditions.

In combination, the survival data suggests, for the first time, that both *E. coli* and *S.* Typhimurium were able to withstand severe environmental conditions in estuarine bank sediments. Overall, the die-off rates observed in this study ranged from 0.14/day to 1.54/day for *E. coli* and 0.21/day to 1.72/day for *S.* Typhimurium. In three of the four configurations (i.e., CD, PI, CI), die-off rates for the two organisms were similar. A wide range of die-off rates has been reported in soil sediment systems for these organisms [27,40,50,51], and differences have been attributed to both biotic and abiotic factors [11,38,49,52,53,54] that are also encountered in bank sediments. 

This study investigated the influence of different bank sediment scenarios on the survival of two fecal microorganisms in potentially extreme weather conditions with respect to temperature, radiation, and desiccation. Our underlying hypothesis is that if the test organisms survive in this study’s extreme climatic conditions, then they can also survive in the less extreme conditions that exist in many systems year-round. With an absence of previous studies focusing on bank sediments under similar experimental conditions, the results of this study significantly enhance our knowledge of the survival of fecal organisms in estuarine bank sediments. Further, this study demonstrates that, without studying fecal pathogen survival in all environments (estuarine bank sediment in this case), perhaps it could be premature to conclude that *E. coli* is not a good indicator organism. 

## 4. Conclusions

This study presented data on the survival of the faecal indicator organism, *E. coli*, and the human fecal pathogen, *S.* Typhimurium, in estuarine bank sediments. Different degrees of tidal inundation were observed to influence the survival for both test organisms, with periodic inundation, mimicking natural tidal cycles, being the most conducive to persistence. While the simultaneous survival rates for these two organisms were found to be comparable, for the first time, this experiment presents evidence that *S.* Typhimurium may survive for over three weeks in estuarine bank sediments, which could be of critical importance. This, in turn, highlights the potential of bank sediments as a source of viable pathogens to the water column, especially when natural processes and anthropogenic activities cause resuspension. Considering the comparable survival patterns of *E. coli* and *S.* Typhimurium in the contrasting experimental conditions, *E. coli* cannot be excluded from being a reliable indicator of public health risks associated with *S.* Typhimurium in estuarine bank sediments.

## Figures and Tables

**Table 1:** Experimental configurations tested for survival of *E. coli* and *S.* Typhimurium and their properties.

Configuration	No. of Replicates	Inoculation with *E. coli* and *S.* Typhimurium	Extent of Inundation
Continuous desiccation (CD)	3	Yes	Never
Extended desiccation (ED)	3	Yes	Starts on Day 7; periodic (cycles of 12.4 h)
Periodic inundation (PI)	3	Yes	Periodic (cycles of 12.4 h)
Continuous inundation (CI)	3	Yes	Continuous
Non-inoculated (NI)	2	No	Periodic (cycles of 12.4 h)

## Appendix A

### Effect of Autoclaving

Autoclaving was done in this experiment to exclude the interference from predation and competition by native organisms present in natural sediments. However, a combination of very high temperature and pressure kills the naturally occurring microbes in the sediment and releases nutrients into the sediment. Also, some other reports suggest that autoclaving can change sediment’s own nutritional properties [42,43,44]. Therefore, it was essential to check if autoclaving was introducing any bias towards higher or lower survival. Therefore, along with the experimental configurations (Table 1), another set (triplicate) of sediments was augmented with a mixture of *E. coli* and *S.* Typhimurium culture (each at a concentration of 10^9^ g^−1^ wet sediment and hereby named as Nutritionally Augmented) before autoclaving. These sediments were eventually inoculated with both test organisms and were tested along the test configurations as mentioned above (Figure A1).

It was observed in this study that there was no obvious impact of nutritional augmentation on *E. coli* or *S.* Typhimurium survival where additional microorganisms were added to the sediment before autoclaving. This could suggest that the extra nutrition (if at all) available to *E. coli* and *S.* Typhimurium through the autoclaving process did not cause significant bias in the observed die-off rates.

## Appendix B

### Changes in Sediments

**Table A1:** Mean (and relative standard deviations in %) of total phosphorus (TP) and total nitrogen (TN) concentrations of sediment samples.

Time during Experiment	*E. coli* (cfu/g dw)	*S.* Typhimurium (cfu/g dw)	TP (mg/g dw)	TN (mg/g dw)
Original bank conditions—not inoculated, not autoclaved				
Day = 0	540	Nil	0.21 *	0.77 *
Autoclaved, not inoculated				
Day = 0	Nil	Nil	0.18 *	0.78 *
Day = 21 (PI)	Nil	Nil	0.4 (12)	1.4 (5)
Autoclaved, spiked with *E. coli*, *S.* Typhimurim and mixed culture				
Day = 0	**	**	0.20 *	0.77 *
Day = 21 (PI)	**	**	0.4 (2)	1.3 (1)
Autoclaved, spiked with *E. coli*, *S.* Typhimurim				
Day = 0	***	***	0.21 *	0.81 *
Day = 21 (ED)	***	***	0.4 (7)	1.4 (3)
Day = 21 (CI)	***	***	0.4 (12)	1.3 (8)
Day = 21 (CD)	***	***	0.4 (4)	1.4 (0)
Day = 21 (PI)	***	***	0.4 (3)	1.4 (3)

* only one sample analysed, ** data presented in Appendix A, *** data presented in Section 3.2.

**Table A2:** Changes of nutrients in inundating water; means (and relative standard deviations in %) of TP and TN concentration of weekly inflow samples.

Time during Experiment	TP (mg/L)	TN (mg/L)	pH	EC (mS/cm)	Turbidity (NTU)
Fresh Inflow					
	0.13 (37)	0.9 (4)	6.6 (4)	0 (17)	2.14 *
Extended Desiccation					
Day = 7			7.4 (2)	0.5 (8)	
Day = 14			5.8 (6)	1.7 (6)	
Day = 21	0.25 (48)	6.4 (48)	5.7 (7)	1 (38)	8.1 (62)
Non-Inoculated					
Day = 7	0.27 (45)	13 (22)	7.2 (1)	2.5 (20)	
Day = 14	0.17 (42)	4.2 (67)	6.1 (1)	0.3 (6)	
Day = 21			5.8 (10)	2.7 (46)	4.4 (37)
Continuously Inundated					
Day = 7			7.5 (0)	2.4 (10)	
Day = 14			6.7 (2)	0.7 (1)	
Day = 21	0.37 (25)	3.6 (19)	7.1 (0)	0.4 (4)	12.3 (44)
Periodically Inundated					
Day = 7	0.27 (19)	10.83 (19)	6.7 (3)	1.9 (23)	
Day = 14	0.2 (12)	5.8 (9)	6 (9)	0.7 (1)	
Day = 21	0.25 (13)	12.2 (43)	5.8 (11)	1.7 (36)	4.4 (30)
Nutritionally Augmented					
Day = 7	0.25 (43)	10.33 (49)	0.1 (1)	1.8 (46)	
Day = 14	0.23 (11)	7.27 (12)	6.4 (9)	0.9 (23)	
Day = 21			6.3 (14)	1.9 (7)	3.7 (27)

* Only one sample was tested against three for the others.

## References

[1] Ross D.A. (1995). Introduction to Oceanography.

[2] Mallin M.A., Williams K.E., Esham E.C., Lowe R.P. (2000). Effect of human development on bacteriological water quality in coastal watersheds. Ecol. Appl..

[3] Catalao-Dionisio L.P., Joao M., Ferreiro V.S., Fidalgo M.L., Garcia-Rosado M.E., Borrego J.J. (2000). Occurrence of *Salmonella* spp. in estuarine and coastal waters of Portugal. Antonie Van Leeuwenhoek.

[4] Samhan F.A., Kronlein M.R., Fakher U., Kronlein C., Stedtfeld R.D., Hashsham S.A. (2009). Detection and occurrence of indicator organisms and pathogens. Water Environ. Res..

[5] Henry R., Schang C., Chandrasena G.I., Deletic A., Edmunds M., Jovanovic D., Kolotelo P., Schmidt J., Williamson R., McCarthy D. (2015). Environmental monitoring of waterborne *Campylobacter*: Evaluation of the Australian standard and a hybrid extraction-free MPN-PCR method. Front. Microbiol..

[6] Edberg S.C., Allen M.J., Smith D.B., Kriz N.J. (1990). Enumeration of total coliforms and *Escherichia coli* from source water by the defined substrate technology. Appl. Environ. Microbiol..

[7] Odonkor S.T., Ampofo J.K. (2013). *Escherichia coli* as an indicator of bacteriological quality of water: An overview. Microbiol. Res..

[8] Australian Government (2008). Guidelines for Managing Risks in Recreational Water.

[9] Schang C., Lintern A., Cook P.L.M., Osborne C.A., McKinley A., Schmidt J., Coleman R., Rooney G., Henry R., Deletic A. (2016). Presence and survival of culturable *Campylobacter* spp. and *Escherichia coli* in temperate urban estuaries. Sci. Total Environ..

[10] Hood M.A., Ness G.E. (1982). Survival of *Vibrio cholerae* and *Escherichia coli* in estuarine waters and sediments. Appl. Environ. Microbiol..

[11] Venkatramanan S., Ramkumar T., Anithamary I. (2013). Distribution of grain size, clay mineralogy and organic matter of surface sediments from Tirumalairajanar Estuary, Tamilnadu, east coast of India. Arab. J. Geosci..

[12] Solo-Gabriele H.M., Wolfert M.A., Desmarais T.R., Palmer C.J. (2000). Sources of *Escherichia coli* in a Coastal Subtropical Environment. Appl. Environ. Microbiol..

[13] Desmarais T.R., Solo-Gabriele H.M., Palmer C.J. (2002). Influence of soil on fecal indicator organisms in a tidally influenced subtropical environment. Appl. Environ. Microbiol..

[14] US-EPA (2009). Fact Sheet: Final Third Drinking Water Contaminant Candidate List (CCL 3).

[15] OzFoodNet Working Group (2006). Burden and Causes of Foodborne Disease in Australia: Annual Report of the OzFoodNet Network, 2005.

[16] Chandran A., Mohamed Hatha A.A. (2005). Relative survival of *Escherichia coli* and *Salmonella typhimurium* in a tropical estuary. Water Res..

[17] Feng F., Goto D., Yan T. (2010). Effects of autochthonous microbial community on the die-off of fecal indicators in tropical beach sand. FEMS Microbiol. Ecol..

[18] Wanjugi P., Harwood V.J. (2013). The influence of predation and competition on the survival of commensal and pathogenic fecal bacteria in aquatic habitats. Environ. Microbiol..

[19] Vdović N., Obhođaš J., Pikelj K. (2010). Revisiting the particle-size distribution of soils: Comparison of different methods and sample pre-treatments. Eur. J. Soil Sci..

[20] McCarthy D.T. (2008). Modelling microorganisms in urban stormwater. Ph.D. Thesis.

[21] Bratieres K., Fletcher T.D., Deletic A., Zinger Y. (2008). Nutrient and sediment removal by stormwater biofilters: A large-scale design optimisation study. Water Res..

[22] Australia Standard (2005). AS 1289.2.1.1: Methods of Testing Soils for Engineering Purposes—Soil Moisture Content Tests—Determination of the Moisture Content of a Soil-Oven Drying Method (Standard Method).

[23] Roszak D.B., Grimes D.J., Colwell R.R. (1984). Viable but nonrecoverable stage of *Salmonella enteritidis* in aquatic systems. Can. J. Microbiol..

[24] Bogosian G., Sammons L.E., Morris P.J., O’Neil J.P., Heitkamp M.A., Weber D.B. (1996). Death of the *Escherichia coli* K-12 strain W3110 in soil and water. Appl. Environ. Microbiol..

[25] Nguyen H.T.M., Le Q.T.P., Garnier J., Janeau J.L., Rochelle-Newall E. (2016). Seasonal variability of faecal indicator bacteria numbers and die-off rates in the Red River basin, North Viet Nam. Sci. Rep..

[26] Whitcomb B.W., Schisterman E.F. (2008). Assays with lower detection limits: Implications for epidemiological investigations. Paediatr. Perinat. Epidemiol..

[27] Mallmann W.L., Litsky W. (1951). Survival of Selected Enteric Organisms in Various Types of Soil. Am. J. Public Health Nations Health.

[28] Pommepuy M., Guillaud J.F., Dupray E., Derrien A., Le Guyader F., Cormier M. (1992). Enteric bacteria survival factors. Water Sci. Technol..

[29] Pote J., Haller L., Kottelat R., Sastre V., Arpagaus P., Wildi W. (2009). Persistence and growth of faecal culturable bacterial indicators in water column and sediments of Vidy Bay, Lake Geneva, Switzerland. J. Environ. Sci..

[30] Garzio-Hadzick A., Shelton D.R., Hill R.L., Pachepsky Y.A., Guber A.K., Rowland R. (2010). Survival of manure-borne *E. coli* in streambed sediment: Effects of temperature and sediment properties. Water Res..

[31] Burton G.A., Gunnison D., Lanza G.R. (1987). Survival of pathogenic bacteria in various freshwater sediments. Appl. Environ. Microbiol..

[32] Malham S.K., Rajko-Nenow P., Howlett E., Tuson K.E., Perkins T.L., Pallett D.W., Wang H., Jago C.F., Jones D.L., McDonald J.E. (2014). The interaction of human microbial pathogens, particulate material and nutrients in estuarine environments and their impacts on recreational and shellfish waters. Environ. Sci. Process. Impacts.

[33] Perkins T.L., Clements K., Baas J.H., Jago C.F., Jones D.L., Malham S.K., McDonald J.E. (2014). Sediment composition influences spatial variation in the abundance of human pathogen indicator bacteria within an estuarine environment. PLoS ONE.

[34] Gerba C.P., McLeod J.S. (1976). Effects of sediments on the survival of *Escherichia coli* in marine waters. Appl. Environ. Microbiol..

[35] Sinton L.W., Davies-Colley R.J., Bell R.G. (1994). Inactivation of enterococci and fecal coliforms from sewage and meatworks effluents in seawater chambers. Appl. Environ. Microbiol..

[36] Davies-Colley R.J., Donnison A.M., Speed D.J., Ross C.M., Nagels J.W. (1999). Inactivation of faecal indicator micro-organisms in waste stabilisation ponds: Interactions of environmental factors with sunlight. Water Res..

[37] Byappanahalli M.N., Nevers M.B., Korajkic A., Staley Z.R., Harwood V.J. (2012). Enterococci in the Environment. Microbiol. Mol. Biol. Rev..

[38] Carlucci A.F., Pramer D. (1960). An evaluation of factors affecting the survival of *Escherichia coli* in sea water. II. Salinity, pH, and nutrients. Appl. Microbiol..

[39] Korajkic A., McMinn B.R., Shanks O.C., Sivaganesan M., Fout G.S., Ashbolt N.J. (2014). Biotic Interactions and Sunlight Affect Persistence of Fecal Indicator Bacteria and Microbial Source Tracking Genetic Markers in the Upper Mississippi River. Appl. Environ. Microbiol..

[40] Marsh P., Morris N.Z., Wellington E.M.H. (1998). Quantitative molecular detection of *Salmonella typhimurium* in soil and demonstration of persistence of an active but non-culturable population. FEMS Microbiol. Ecol..

[41] Chen Z., Diao J., Dharmasena M., Ionita C., Jiang X., Rieck J. (2013). Thermal Inactivation of Desiccation-Adapted *Salmonella* spp. in Aged Chicken Litter. Appl. Environ. Microbiol..

[42] Begley M., Hill C. (2015). Stress adaptation in foodborne pathogens. Annu. Rev. Food Sci. Technol..

[43] Frey S.K., Gottschall N., Wilkes G., Grégoire D.S., Topp E., Pintar K.D.M., Sunohara M., Marti R., Lapen D.R. (2015). Rainfall-induced runoff from exposed streambed sediments: An important source of water pollution. J. Environ. Qual..

[44] An Y.J., Kampbell D.H., Breidenbach G.P. (2002). *Escherichia coli* and total coliforms in water and sediments at lake marinas. Environ. Pollut..

[45] Grimes D.J. (1975). Release of Sediment-Bound Fecal Coliforms by Dredging. Appl. Microbiol..

[46] D’Aoust J.Y., Maurer J., Doyle M.P., Beuchat L.R. (1997). *Salmonella* species. Food Microbiology: Fundamentals and Frontiers.

[47] Stephenson G.R., Rychert R.C. (1982). Bottom Sediment: A Reservoir of *Escherichia coli* in Rangeland Streams. J. Range Manag..

[48] Chudoba E.A., Mallin M.A., Cahoon L.B., Skrabal S.A. (2013). Stimulation of fecal bacteria in ambient waters by experimental inputs of organic and inorganic phosphorus. Water Res..

[49] Ferguson C.M., Coote B.G., Ashbolt N.J., Stevenson I.M. (1996). Relationships between indicators, pathogens and water quality in an estuarine system. Water Res..

[50] Tate R.L. (1978). Cultural and Environmental Factors Affecting the Longevity of *Escherichia coli* in Histosols. Appl. Environ. Microbiol..

[51] Zibilske L.M., Weaver R.W. (1978). Effect of Environmental Factors on Survival of *Salmonella typhimurium* in Soil1. J. Environ. Qual..

[52] Fujioka R.S., Hashimoto H.H., Siwak E.B., Young R.H. (1981). Effect of sunlight on survival of indicator bacteria in seawater. Appl. Environ. Microbiol..

[53] Crane S.R., Moore J.A. (1986). Modeling enteric bacterial die-off: A review. Water Air Soil Pollut..

[54] Semenov A.V., van Overbeek L., Termorshuizen A.J., van Bruggen A.H. (2011). Influence of aerobic and anaerobic conditions on survival of *Escherichia coli* O157:H7 and *Salmonella enterica* serovar Typhimurium in Luria-Bertani broth, farm-yard manure and slurry. J. Environ. Manag..

